# The Effects of Photobiomodulation on Bone Defect Repairing in a Diabetic Rat Model

**DOI:** 10.3390/ijms222011026

**Published:** 2021-10-13

**Authors:** Ji-Hua Lee, Su-Chii Kong, Chia-Hsin Chen, Ying-Chun Lin, Kun-Tsung Lee, Yan-Hsiung Wang

**Affiliations:** 1 School of Dentistry, College of Dental Medicine, Kaohsiung Medical University, Kaohsiung 807387, Taiwan; u99820003@kmu.edu.tw; 2 Orthopaedic Research Center, College of Medicine, Kaohsiung Medical University, Kaohsiung 807387, Taiwan; sckong2015@gmail.com (S.-C.K.); chchen@cc.kmu.edu.tw (C.-H.C.); 3 Division of Medical Biochemistry, University of Cape Town, Rondebosch, Cape Town 7701, South Africa; 4 Department of Physical Medicine and Rehabilitation, School of Medicine, College of Medicine, Kaohsiung Medical University, Kaohsiung 807387, Taiwan; 5 Department of Physical Medicine and Rehabilitation, Kaohsiung Medical University Hospital, Kaohsiung 807387, Taiwan; 6 Department of Oral Hygiene, College of Dental Medicine, Kaohsiung Medical University, Kaohsiung 807387, Taiwan; bonnie0925.tw@gmail.com; 7 Department of Dentistry, Kaohsiung Medical University Hospital, Kaohsiung Medical University, Kaohsiung 807387, Taiwan; 8 Department of Medical Research, Kaohsiung Medical University Hospital, Kaohsiung 807387, Taiwan; 9 Regenerative Medicine and Cell Therapy Research Center, Kaohsiung Medical University, Kaohsiung 807387, Taiwan

**Keywords:** photobiomodulation therapy, diabetic mellitus, bone defect repair

## Abstract

The purpose of this study is to examine the prospective therapeutic effects of photobiomodulation on the healing of bone defects in diabetic mellitus (DM) using rat models to provide basic knowledge of photobiomodulation therapy (PBMT) during bone defect repair. For in vitro study, an Alizzarin red stain assay was used to evaluate the effect of PBMT on osteogenic differentiation. For in vivo study, micro-computed tomography (microCT) scan, H&E and IHC stain analysis were used to investigate the effect of PBMT on the healing of the experimental calvarial defect (3 mm in diameter) of a diabetic rat model. For in vitro study, the high glucose groups showed lower osteogenic differentiation in both irradiated and non-irradiated with PBMT when compared to the control groups. With the PBMT, all groups (control, osmotic control and high glucose) showed higher osteogenic differentiation when compared to the non-irradiated groups. For in vivo study, the hyperglycemic group showed significantly lower bone regeneration when compared to the control group. With the PBMT, the volume of bone regeneration was increasing and back to the similar level of the control group. The treatment of PBMT in 660 nm could improve the bone defect healing on a diabetic rat calvarial defect model.

## 1. Introduction

Diabetes mellitus (DM) is a chronic metabolic impairment leading to hyperglycemia developed from relative or absolute insulin deficiency. There are several forms of DM, i.e., Type 1 diabetes mellitus (T1DM), Type 2 diabetes mellitus (T2DM), gestational diabetes, and other specific forms [1]. T1DM is the major type of DM in youth resulting from a destruction of the pancreatic β-cells of autoimmune etiology, leading to insulin deficiency [2,3]. T2DM is much more common affecting 90% of all diabetes patients. It is characterized by insulin resistance coupled with relative insulin insufficiency and an excessive or inappropriate glucagon secretion [4,5]. As the glucose levels are elevated, it contributes to many complications seen in DM, including skeletal disorders known as diabetic osteopathy accompanied by reduced bone quality in patients [6].

Both T1DM and T2DM are associated with diabetic osteopathy with increased risk for bone fracture and delayed fracture reunion as evidenced by meta-analyses and cohort studies, although the risk of fractures has been reported to be significantly greater in T1DM when compared to T2DM patients [7,8,9,10]. Several distinctive features can be identified in the diabetic osteopathic phenotype: (i) diminished linear bone growth during the pubertal growth spurt in adolescents with diabetes [11]; (ii) reduction of bone mineral density and increased risk for occurrence of osteopenia and osteoporosis [10]; (iii) increased fracture risk [12]; (iv) poor osseous healing characteristics and impaired bone regeneration potential [13]. Previous studies indicated that hyperglycemia contributes to these phenotypes and later affects the bone healing process in several ways. Hyperglycemic state in DM may affect the skeletal tissue metabolism, induce apoptosis in osteoblasts, chondrocytes and endothelial cells, reduce osteogenic differentiation, increase osteoclast activity, and bring the immune system to a pro-inflammatory state, leading to delayed bone healing [14,15,16]. However, the exact underlying molecular mechanisms are hitherto poorly defined. The lack of understanding leads to the lack of the effective therapeutic interventions and strategies in bone fracture repair in DM at the present time.

Photobiomodulation therapy (PBMT) consists of non-thermal irradiation at wavelengths between visible light and the near-infrared range [17]. The non-destructive energy exerted by PBMT stimulates a series of photobiological reactions reported to be beneficial for therapeutic purposes by the principle of biomodulation, i.e., using the native raw materials produced within our body to produce favorable changes in tissues [18]. Several cellular signaling pathways were triggered by PBMT application to promote cell growth, survival, proliferation, collagen synthesis, and differentiation [19,20,21,22]. In vitro, it has shown to be favorable on bone fracture repair by increasing mitochondrial activity thus ATP synthesis, DNA/RNA synthesis in osteoblasts, cell proliferation, cell viability and the expression of alkaline phosphatase (ALP) [23,24,25,26]. Additionally, PBMT has been shown to enhance bone regeneration in vivo by upregulating cyclooxygenase-2, enhancing endochondral ossification, increasing bone density and bone matrix formation, and inducing osteoblast activity and vascularization [27,28,29,30,31,32]. Our previous study has demonstrated that PBMT in 660 nm was able to suppress the inflammatory reaction implied in many diabetic complications [33,34]. This observation is in agreement with the findings reported by Pinheiro et al., whereby light emitting diode (LED) phototherapy reduced inflammation and increased both collagen and bone deposition [35].

This present study aimed to study the prospective therapeutic effects of PBMT on the healing of bone defects in DM rats by microCT images and histomorphological analysis. Additionally, we further provide basic knowledge of PBMT during bone defect repair on diabetic hyperglycemia.

## 2. Results

### 2.1. Higher Calcium Deposition of D1 Cells Cultured in High Glucose Medium Was Observed after PBMT Treatment

**Figure 1:**
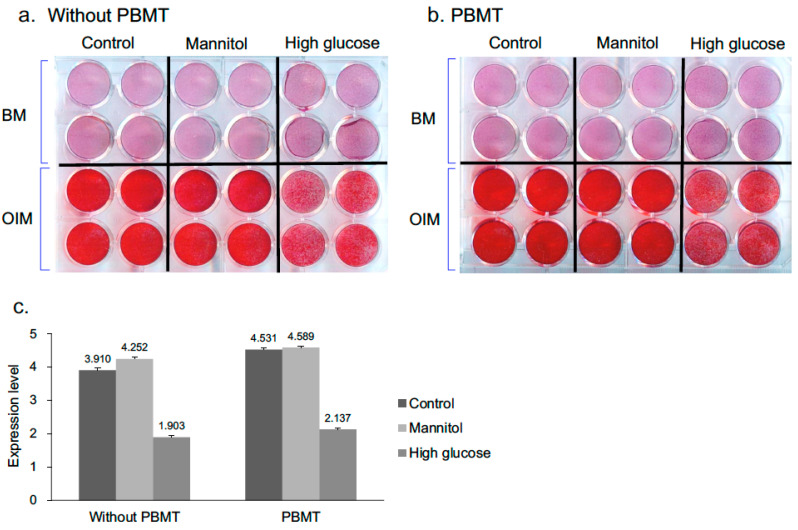
Analysis of the mineral deposition in D1 cells on day 7 by Alizarin Red S staining. (**a**,**b**) Representative photographs from Alizarin Red S staining assays. D1 cells were cultured in BM or OIM with low or high glucose content for 7 days. PBMT treatment (8 J/cm^2^) was applied daily directly onto cells. Cells were stained with Alizarin Red S for mineralization analysis. (**c**) Quantitative data obtained from destained Alizarin red S absorbance. The results are expressed as means ± SD of three replicates.

Alizarin Red S staining, which stains deposited calcium red, was carried out to assess the osteogenic differentiation of the D1 cells. When the cells were cultured in osteogenic induction medium (OIM) over a period of 7 days, osteogenic differentiation was showed and cells were stained positive with Alizarin Red S (Figure 1a, lower panel on plate). Conversely, the cells cultured in the medium without osteogenic induction reagents (bone medium, BM) were negatively stained with Alizarin Red S (Figure 1a, upper panel on plate) on day 7. The cells cultured in both normal and osmotic control mannitol media were stained strongly by Alizarin Red S post-osteogenic induction; while cells cultured in hyperglycemic condition were stained weakly (Figure 1a,b). Upon PBMT treatment, the mineralization capacity was increased in cells cultured in all conditions as compared to their corresponding groups without PBMT treatment when assessed quantitatively (Figure 1c).

### 2.2. PBMT Treatment Increased the Healing of DM Calvarial Bone Defects

**Figure 2:**
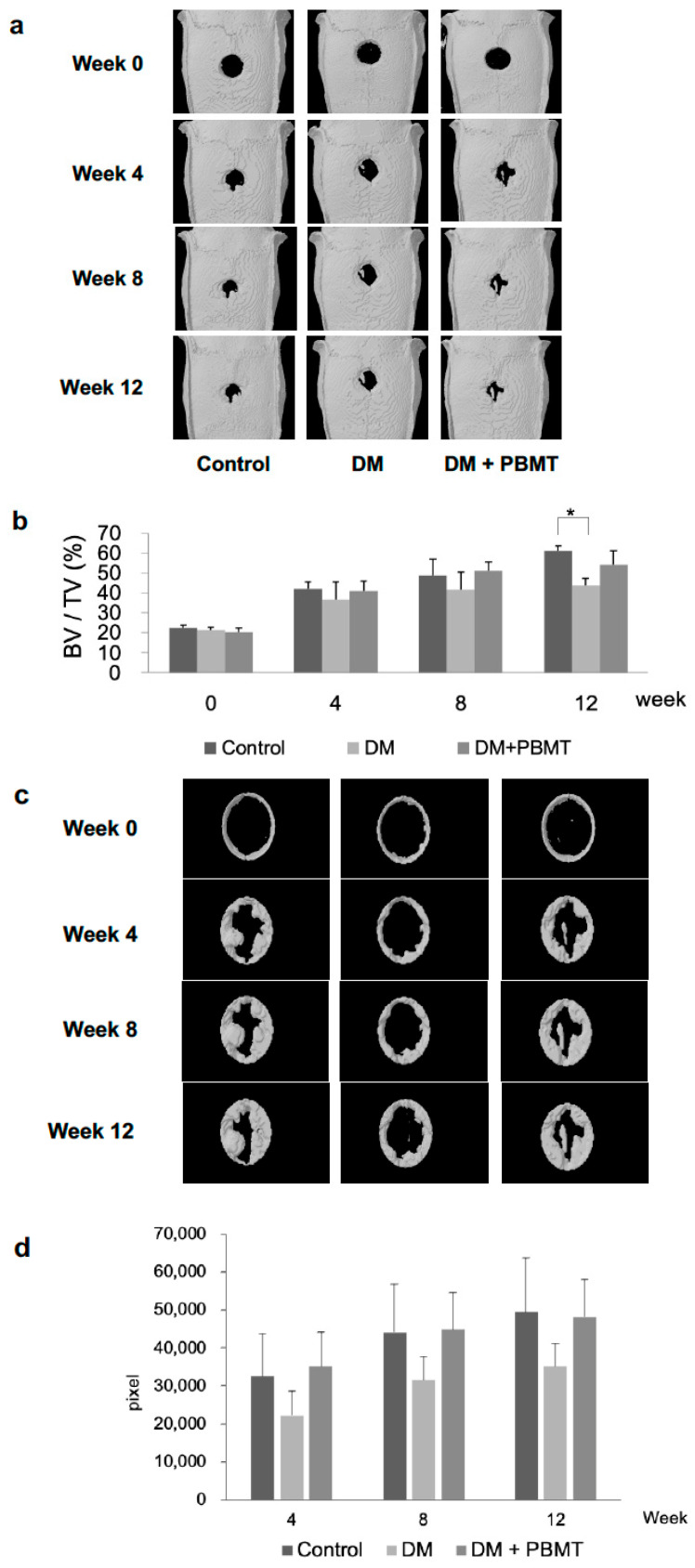
MicroCT analysis of in vivo calvarial bone repair at week 12 post-operation. (**a**) Representative microCT images of calvarial bone sections from each group: Control, DM, and DM + PBMT treatment. Calvarial bone defects (3 mm) were generated in adult Wistar rats and PBMT treatment (4 J/cm^2^) was applied daily. The follow-up imaging and analyses were performed at Weeks 0, 4, 8, 12 postoperatively to quantify residual defect volume and new bone formation. (**b**) Quantitative measurements obtained from microCT images. The data illustrated as the percentage of bone volume (BV) per total volume (TV) of the calvarial defect and expressed as means ± SD, * *p* < 0.05. (**c**) Representative microCT images of calvarial bone matrix formation at Weeks 0, 4, 8 and 12 from each group: Control, DM, and DM + PBMT treatment. Calvarial bone defects (3 mm) were generated in adult Wistar rats and PBMT treatment (4 J/cm^2^) were applied daily. The close-up imaging on the calvarial defect sites were performed to evaluate new bone matrix formation at Weeks 0, 4, 8, 12 postoperatively. (**d**) Quantitative measurements obtained from microCT images of the calvarial bone matrix formation at Weeks 0, 4, 8 and 12. The data is illustrated as the density measurements from the pixel mapping of the region of interest (ROI) and expressed as means ± SD.

The microCT was performed at Weeks 0, 4, 8, and 12 post-operation to assess bone regeneration. Representative 3D microCT scans are shown in Figure 2a. After 4 weeks of healing, marginal bone formation occurred from the margin of the defects in all control and experimental groups. The amount of newly formed bone was higher in the non-treatment normal non-diabetic group (Normal group) at any time point (except Week 0). The DM group showed significant reductions of new bone formation in calvarial bone defect as compared to the control group at Week 12 postoperatively. After the healing occurred at Weeks 4, 8, and 12, bone formation could be seen as higher in the DM + PBMT group as compared to the DM group and no statistically significant increment of bone formation was detected in the DM + PBMT group when compared to the DM group (Figure 2b).

A close-up microCT analysis was carried out to further assess the new bone matrix formation area at Weeks 0, 4, 8, and 12 post-operation from the calvarial defect sites. Representative scanned images of the close-up measurements of the new bone matrix formation are demonstrated in Figure 2c. The results revealed that the bone formation in the DM group could be seen lower as compared to the non-treatment normal non-diabetic group (Normal group) and the DM + PBMT group (Figure 2c,d) after 4 weeks of bone healing. Similar observations were sustained at Weeks 8 and 12. The new bone matrix formation after PBMT treatment (DM + PBMT group) was seen higher than the DM group without treatment at the end point of the experiment (Week 12).

**Figure 3:**
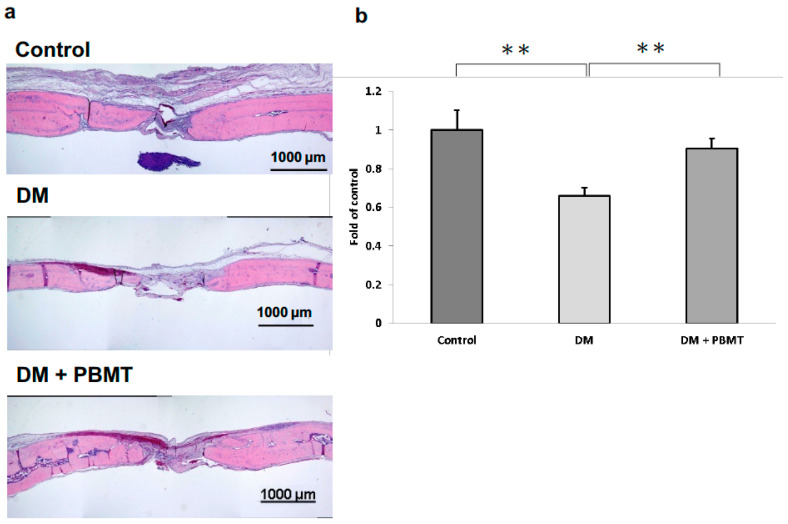
H&E staining of the cross section of the calvarial defects at Week 12 post-operation. (**a**) Representative H&E staining images of calvarial bone sections from each group: Control, DM, and DM + PBMT treatment. Calvarial bone defects (3 mm) were generated in adult Wistar rats. PBMT treatment (4 J/cm^2^) were applied daily. The calvarial bone sections were collected at Week 12 postoperatively and stained using H&E. Scale bar, 1000 μm. (**b**) Quantitative measurements obtained from H&E-stained images. The new bone formation area in the calvarial defect site was measured and the percentage of bone matrix within the callus was calculated. Data were expressed as means ± SD, ** *p* < 0.001.

Next, H&E staining was performed at Week 12 post-operation to examine the quality of regenerated bone at the calvarial defect sites. Representative images of the H&E-stained cross section of the calvarial defects are shown in Figure 3a. Complete repair of the defects was not observed in all groups. The center of the defects from all the groups was detected to have filled with thin, loose connective tissue (Figure 3a). Confirming the microCT observations, the normal group showed the most new bone formation originating from the defect margins as compared to the experimental groups. In addition, the normal group showed significant bone regeneration as compared to the DM group. Moreover, the bone formation of DM rats was significantly increased after the PBMT treatment at Week 12 when assessed quantitatively by computer-assisted histometric analysis (Figure 3b).

### 2.3. No Increase in Osteogenic Factor BMP-2 Expression Was Detected upon PBMT Treatment at Week 12

**Figure 4:**
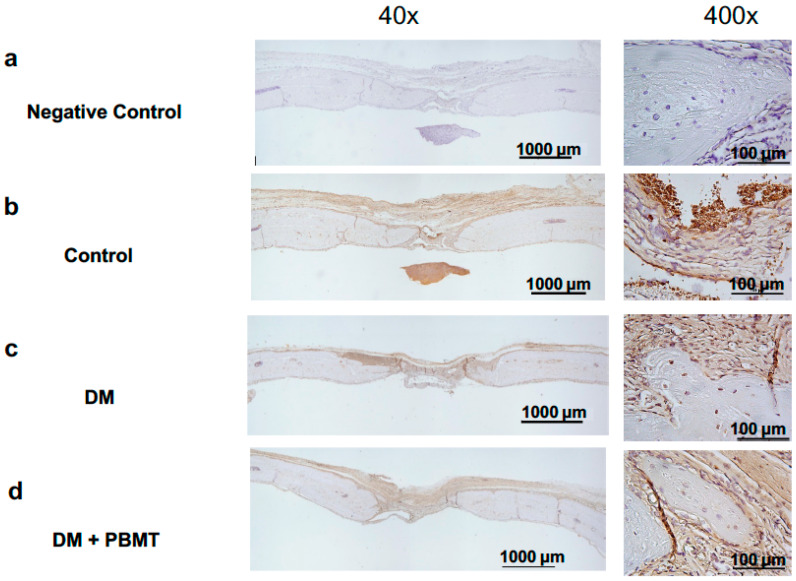
IHC staining of osteogenic factors BMP-2 of the cross section of the calvarial defects at Week 12 post-operation. Representative BMP-2-stained images of calvarial bone sections from each group: Non-antibody staining control (**a**), Control (**b**), DM (**c**), and DM + PBMT treatment (**d**). Calvarial bone defects (3 mm) were generated in adult Wistar rats. PBMT treatment (4 J/cm^2^) was applied daily. The calvarial bone sections were collected at Week 12 postoperatively and stained with BMP-2 antibodies. Scale bar, 1000 μm for 40× and 100 μm for 400×.

IHC was applied to detect the expression levels of osteogenic factor BMP-2 at Week 12 post-operation. Representative images of the BMP-2-stained cross section of the calvarial defects are shown in Figure 4. The result showed no detectable difference of BMP-2 expression between the normal (Figure 4b) and experimental samples (Figure 4c,d), with or without PBMT treatment, collected at the experiment end-point (Week 12; Figure 4). 

## 3. Discussion

Numerous studies have previously demonstrated that PBMT treatment promotes accelerated bone regeneration and bone healing [36,37,38,39,40,41,42]. However, only a few studies have focused on the biostimulative effect of PBMT on bone repair under hyperglycemia condition. In this study, the in vitro experiments showed that PBMT increased the calcium depositions on D1 cells cultured under high glucose condition (Figure 1), indicating an increased osteogenic differentiation of the cells into mature osteoblasts for bone formation. Hendudari et al. reported that PBMT increased the survival of high glucose cultured human dermal fibroblast [43]. Esmaeelinejad et al. also reported that PBMT increased the proliferation rate and cell viability of human skin fibroblasts at both physiologic and high glucose levels [44]. These studies are consistent with our current study which indicated that PBMT possesses the potential ability to facilitate the bone healing undergoing hyperglycemia. 

Yildirimturk S et al. reported that PBMT application promoted vascularization and new bone formation in animals with DM to a limited extent since it was unable to support the healing process up to the level of non-diabetic animals [45]. In our study, the results showed an enhancement in the healing of the DM rat defected calvarial bone after PBMT treatment over the span of 12 weeks via both in vivo microCT image analyses (Figure 2) and histomorphometric analysis (Figure 3). Although we did not observe a complete healing of the calvarial defects, the defects were much smaller compared to the original defect. We anticipate that a complete healing will be accomplished in a longer period of time. Our results are consistent with Yildirimturk’s study although Yildirimturk only observed a limited healing in the DM + PBMT group which is probably due to the short interval to observe in four weeks. 

BMP-2 is involved in the induction of the differentiation of osteogenic stem cells into osteoblasts, thus an important regulating factor during osteogenesis [46]. Wu and colleagues have suggested that the osteogenic differentiation of these cells after PBMT treatment may have occurred through the BMP-2 and IGF1 signaling pathways [17]. We also assessed the expression level of BMP-2 on collected calvarial samples in the current studies. Upon IHC staining, no difference in the expression level of BMP-2 was detected among all experimental groups. Since the samples were collected at the post-12 week experimental end-point, the BMP-2 expressions of each group might have reached plateau and showed no differences. Future studies should assess the quantification of mRNA expression levels of this protein at different critical time points throughout the bone healing process after the PBMT treatment.

The optimal energy density of the laser biostimulation effect is still under debate. Parenti et al. noted that the viability, DNA content, and the release of VEGF of Saos-2 human osteoblast-like cells were increased by laser irradiation in a dose-dependent manner [47]. The parameter setting of 660 nm PBMT on cell proliferation and differentiation is about 4 J/cm^2^ in several studies [48,49,50,51,52] and only few studies investigated the biostimulatory effect at higher energy density [53,54]. In this present study, different energy density of PBMT was applied, respectively, in the in vitro (8 J/cm^2^) and in vivo (4 J/cm^2^) experiments, in which a lower energy density (4 J/cm^2^) was applied in the in vivo experiments. We investigated the biostimulatory effect of a 660 nm laser at 8 J/cm^2^ on cell proliferation and differentiation of mBMSCs. The results showed that laser with 8 J/cm^2^ resulted in a positive effect on mBMSCs in a hyperglycemia condition. In the in vivo experiments, if the time length required for the irradiation was set correspondingly to in vitro experiments, the irradiation time would be too extensive for the experimental animals during the process, and the experiment protocol would be ineffective in time and cost. In the future, the effect of higher energy density of PBMT is worth studying with the improvement of laser devise energy output.

## 4. Materials and Methods

### 4.1. Cell Culture

The mouse bone marrow mesenchymal stem cells (mBMSCs, or D1 cells) were purchased from ATCC Cells. They were cultured in the bone medium (BM; DMEM supplemented with 10% fetal bovine serum (Life Technologies, Inc., Carlsbad, CA, USA), 100 U/mL of penicillin, 100 mg/mL of streptomycin, and 2.2 g/L sodium carbonate (Thermo Fisher Scientific, Waltham, MA, USA)). Cells were divided into three groups: vehicle (Control), 30 mM D-mannitol (Osmotic control), or 30 mM D-glucose (High Glucose). Culture medium was replaced with osteoinduction media (OIM; culture media supplemented with 10 nM dexamethasone, 20 µM β-glycerol phosphate, and 50 µM 1-ascorbic acid 2-phosphate) to induce osteogenic differentiation and changed 3 times a week before cells were collected for alizarin red S staining.

### 4.2. Laser Irradiation

A gallium-aluminum-arsenide (GaAIAs) red laser (wavelength 660 nm; Transverse Ind. Co. Ltd., Taipei, Taiwan) was applied as the light source. In in vitro experiments, the distance between the laser source and the cell surface was 3 cm to match the growth area (3.96 cm^2^) of a 12-well culture plate. The cells were irradiated daily on a clean bench at room temperature for 528 s to receive 8 J/cm^2^ of laser energy density. For the undermentioned in vivo experiments, the distance between the laser source and the vertex of rat was 2 cm for the spot size of the laser beam (2.64 cm^2^) to fully cover the defected area of the calvarial bone. The calvarial bone defect site of the rat was irradiated for 1652 s to receive 4 J/cm^2^ of laser energy density taking into account the diminished energy density on the penetration of skin tissue. Laser treatment was applied daily for 12 weeks and the animals were euthanized with anesthetic associated with relaxant for sample collection.

### 4.3. Alizarin Red S Staining

Alizarin Red S staining was performed to assess the osteogenic differentiation of the cells cultured in osteoinduction medium and with laser treatment. Briefly, mediums were removed and the cells were washed gently with PBS, fixed with 10% formaldehyde for 15 min then were removed and dried at room temperature for 10 min. Alizarin Red S staining solution (1 g/100 mL in ddH_2_0; Sigma-Aldrich, St. Louis, MO, USA), a negatively charged dye for the detection of mineralized matrix, was added and incubated at room temperature for 15 min. The staining solution was then discarded and the cells were washed 3 times with distilled water to eliminate nonspecific staining, air-dried and photographed. Later, 10% acetic acid was added and incubated at 60 °C for 30 min. The dissolved solution was collected, and the absorbance was read at 490 nm with a microplate reader.

### 4.4. Experimental Design

In total, 15 specific pathogen-free 8-week-old male Wistar rats (250–275 g) were purchased from LASCO (Bio Lasco Taiwan Co., Ltd., Taipei, Taiwan) and housed under standard laboratory conditions with food and water provided ad libitum. The animals were divided into three groups: control non-diabetic group (Normal), diabetic group (DM), or diabetic group with PBMT treatment (DM + PBMT). Induction of diabetes (hyperglycemia) was accomplished by a single intraperitoneal injection of streptozotocin (STZ, 60 mg/kg, dissolved in citrate buffer, pH 4.5) into rats. The fasting blood glucose level was measured 10 days after STZ injection. Diabetes is defined by a fasting blood glucose exceeding 200 mg/dL. All animals were handled according to the guidelines approved by the Animal Care and Use Committee of Kaohsiung Medical University, Kaohsiung, Taiwan.

### 4.5. Experimental Calvarial Defect

The 3 mm in diameter calvarial defects were made in the right parietal bone of adult male Wistar rats (n = 5 for each group). The exposed skin over the scalp was incised sagittal before a 3 mm full-thickness defect was made using a dental surgical drilling unit with a trephine as previously mentioned [55]. The defect site was later rinsed with saline to wash out any bone fragments. Animals were taken in vivo microCT at Weeks 0, 4 and 8 then sacrificed after 12 weeks, respectively, for subsequent microCT observation and histological analyses. All animals survived the surgical procedures in terms of morbidity and mortality. No visible complications such as infection or suppuration were observed throughout the experimental period. No complete post-operative bone healing was observed on all the calvarial bone defects generated.

### 4.6. Micro-Computed Tomography (Microct)

The animals were scanned using a high-resolution microtomograph device (Skyscan, Kontich, Belgium) at a resolution of 35 μm and with the settings 50 kV and 200 μA. For the fracture healing experiments, the region of interest (ROI) is defined as the area of calvarial defects (3 mm in diameter). In all ROIs, the total tissue volume and the bone volume fraction (BV/TV) were calculated. Furthermore, the region was narrowed down for a close-up measurement of new bone matrix formation with CT-volume (CTVol; Skyscan, Kontich, Belgium) application. The quantification was carried out using Image-Pro Plus 5.0 software.

### 4.7. Histomorphological Analysis and Immunostaining of Bone Tissue

All bone tissue samples were decalcified using 0.5 M ethylenediaminetetraacetic acid (EDTA) in double-distilled water, followed by fixation with 4% paraformaldehyde. After paraffin wax embedding, the samples were divided into frontal and rear portions according to the middle of the calvarial defect in each sample. For each side, serial 5 μm sections were obtained sequentially from the mid-line to the edge, and 15 sections were collected from each side. The sections were routinely processed with H&E staining and observed using a microscope. The new bone formation area in calvarial defect site was measured using Image-Pro Plus 5.0 software (Media Cybernetics Inc., Rockville, MD, USA). The percentage of bone matrix within the callus was calculated. IHC staining of osteogenic factors BMP-2 were carried out next. In brief, after a series of dewaxing and rehydration of paraffin sections with xylene and a series of decreasing alcohol concentrations, the sections were treated with 1 mg/mL pronase (Sigma-Aldrich, St. Louis, MO, USA) for 60 min at 37 °C for antigen retrieval. The sections were then incubated overnight at 4 °C with a 1:1000 dilution of polyclonal rabbit BMP-2 antibody. Secondary antibodies goat anti-rabbit biotinylated immunoglobulin (Abcam, Cambridge, UK) were applied at a 1:1000 dilution for 2 h at room temperature. An avidin–biotin–peroxidase complex (DakoCytomation, Glostrup, Denmark) was applied at a 1:1000 dilution for 40 min at 37 °C. The peroxidase activity was detected using 0.4 mg/L of 3.3′-diaminobenzidine (DAB solution) in phosphate buffer at pH 7.3 in the presence of 0.12% H_2_O_2_. The sections were then counterstained with hematoxylin. The signals of immunostaining were also measured using Image-Pro Plus 5.0 software.

### 4.8. Statistical Analysis

SPSS version 17.0 was used for the statistical analysis. All results were expressed as the mean ± standard deviation. Statistically significant differences between the control and experimental groups were determined using the Student’s *t*-test and analysis of variance (ANOVA) followed by a post hoc Tukey’s test for multiple comparisons. A *p*-value less than 0.05 is considered as statistically significant.

## 5. Conclusions

In this study, our data showed that PBMT treatment of 660 nm enhanced osteogenic activity of BMSCs in both normal and hyperglycemic conditions. Furthermore, we demonstrated that PBMT could improve the bone fracture healing on a diabetic rat calvarial defect model. PBMT may be used as a potential clinical treatment to improve bone healing in DM patients.

## Data Availability

The data presented in this study are available on request from the corresponding author within the framework of a scientific cooperation.
